# Ultrasound radiomics model-based nomogram for predicting the risk Stratification of gastrointestinal stromal tumors

**DOI:** 10.3389/fonc.2022.905036

**Published:** 2022-08-26

**Authors:** Minling Zhuo, Jingjing Guo, Yi Tang, Xiubin Tang, Qingfu Qian, Zhikui Chen

**Affiliations:** Department of Ultrasound, Fujian Medical University Affiliated Union Hospital, Fuzhou, China

**Keywords:** gastrointestinal stromal tumors, radiomics, ultrasound, risk grade, model, nomogram

## Abstract

This study aimed to develop and evaluate a nomogram based on an ultrasound radiomics model to predict the risk grade of gastrointestinal stromal tumors (GISTs). 216 GIST patients pathologically diagnosed between December 2016 and December 2021 were reviewed and divided into a training cohort (n = 163) and a validation cohort (n = 53) in a ratio of 3:1. The tumor region of interest was depicted on each patient’s ultrasound image using ITK-SNAP, and the radiomics features were extracted. By filtering unstable features and using Spearman’s correlation analysis, and the least absolute shrinkage and selection operator algorithm, a radiomics score was derived to predict the malignant potential of GISTs. a radiomics nomogram that combines the radiomics score and clinical ultrasound predictors was constructed and assessed in terms of calibration, discrimination, and clinical usefulness. The radiomics score from ultrasound images was significantly associated with the malignant potential of GISTs. The radiomics nomogram was superior to the clinical ultrasound nomogram and the radiomics score, and it achieved an AUC of 0.90 in the validation cohort. Based on the decision curve analysis, the radiomics nomogram was found to be more clinically significant and useful. A nomogram consisting of radiomics score and the maximum tumor diameter demonstrated the highest accuracy in the prediction of risk grade in GISTs. The outcomes of our study provide vital insights for important preoperative clinical decisions.

## Introduction

Gastrointestinal stromal tumors (GISTs) are the most common mesenchymal-derived tumors of the gastrointestinal tract, with diverse biological behaviors (1, 2). Preoperative prediction of the malignant potential of GISTs is of great significance for clinical treatment and prognostic prediction (3, 4). However, the preoperative assessment of the risk grading of gastrointestinal stromal tumors is difficult because of the difficulty in calculating mitotic counts preoperatively. Therefore, the research on identifying a preoperative diagnostic method that is reproducible and can objectively predict the risk grading of GISTs has recently attracted significant attention in in recent years.

The transabdominal ultrasonography, especially medium- and high-frequency ultrasonography, can clearly display the layers of the gastrointestinal wall. It can be easily operated and can be dynamically observed repeatedly. The transabdominal gastrointestinal ultrasonography is a commonly used method in preoperative imaging examination of GIST. Although transabdominal ultrasonography can directly evaluate the tumor size, location, shape, boundary, echo homogeneity, and blood flow signals, which is of great value for tumor detection, as well as the localization and diagnosis, this method was easily affected by examiner’s subjective vision and diagnostic experience. Thus, because of tumor heterogeneity, these subjective assessments have relatively low accuracy and repeatability (5, 6).With the development of artificial intelligence, radiomics prediction models have gained attention in cancer diagnosis (7–10). Radiomics can extract inaccessible feature data from medical images with a high-throughput and has great application prospects in predicting the biological behavior of tumors (11, 12).

In recent years, some studies have explored the value of radiomics based on computed tomography or magnetic resonance imaging to predict the malignant potential of GISTs (13, 14); however, the application value of ultrasound has not been reported. In this study, accessible and universal ultrasound images were selected as the basic imaging data, and the internal characteristic information of the tumors was extracted. In order to integrate radiomics features, clinical factors, and conventional ultrasound features to adequately evaluate and effectively support preoperative clinical management, we aimed to construct a radiomics nomogram to predict the risk grade of GISTs in this study.

## Materials and methods

### Study population

**Figure 1:**
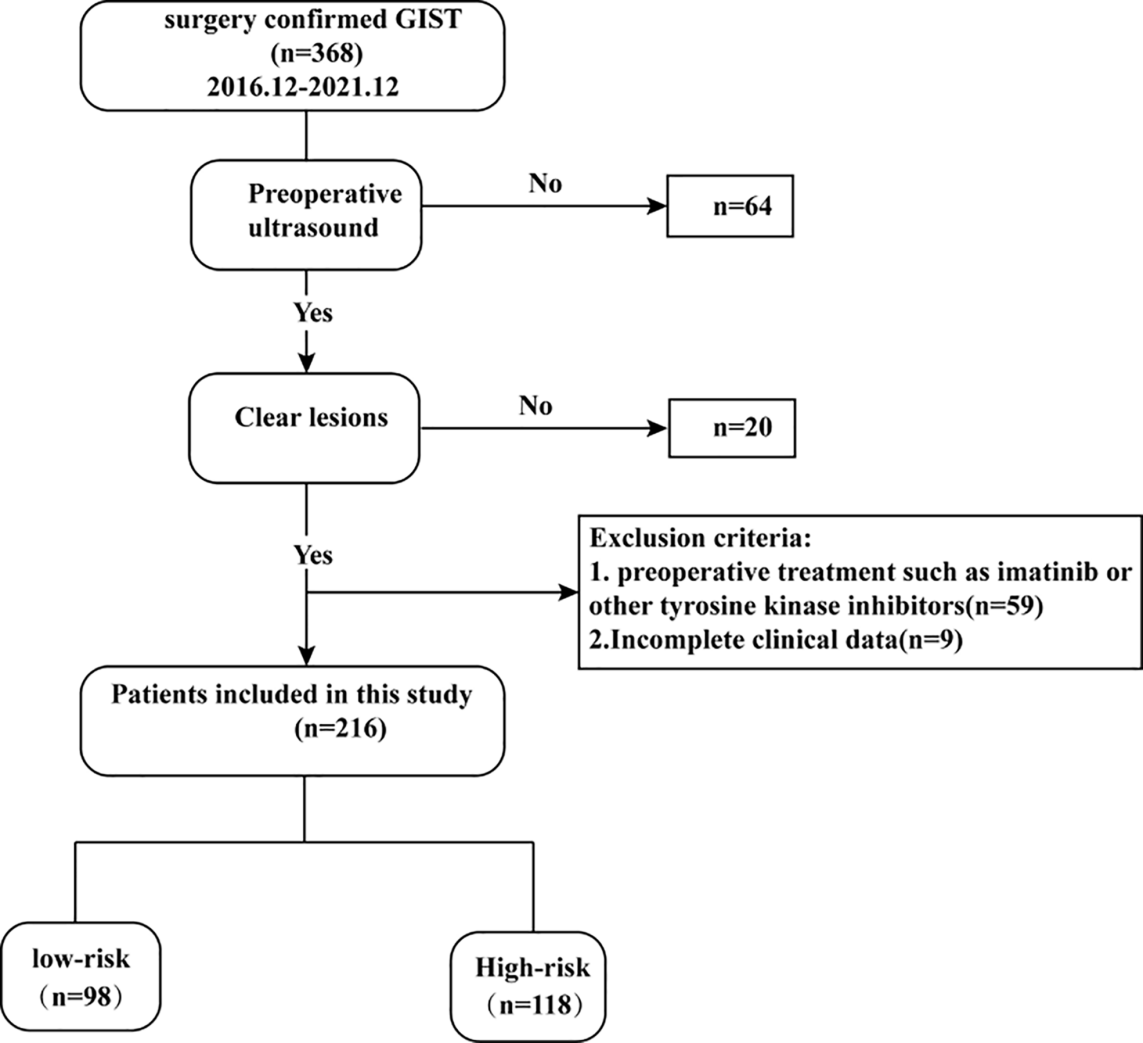
Flowchart of inclusion and exclusion of the study population.

This retrospective study was approved by the ethics committee of Fujian Medical University Affiliated Union Hospital. The signed informed consent forms were waived. From December 2016 and December 2021, 368 GIST patients histologically confirmed at our institution were retrospectively recruited. The inclusion criteria were as follows: 1) the diagnosis of GISTs was confirmed by postoperative pathology, 2) the patients performed ultrasonic examination within 15 days before operation, and 3) the ultrasound image clearly showed the target lesion. The excluding standards were as follows: 1) patient received other preoperative treatment such as imatinib or other tyrosine kinase inhibitors and 2) incomplete ultrasound and clinical data. In this study, 216 patients were enrolled (126 men and 90 women; mean age 60 ± 11 years; range 28–86 years) (**Figure 1**). Patients were divided into a training cohort (n = 163; 95 men and 68 women; mean age 60 ± 12 years) and validation cohort (n = 53; 31 men and 22 women; mean age 59 ± 11 years) after simple randomization at a ratio of 3:1. According to the pathological results and the National Institutes of Health modified criteria, patients in this study were divided into low-malignant potential (very low to low risk) and high-malignant potential (intermediate to high risk) groups.

### Clinical characteristics

Preoperative demographic and clinicopathological data of the 216 GIST patients were collected, including age, gender, height, weight, tumor location (stomach or extragastric), and tumor size (maximum diameter). In addition, this study collected postoperative data, including the risk of NIH stratification and growth patterns.

### Radiomics analysis process

**Figure 2:**
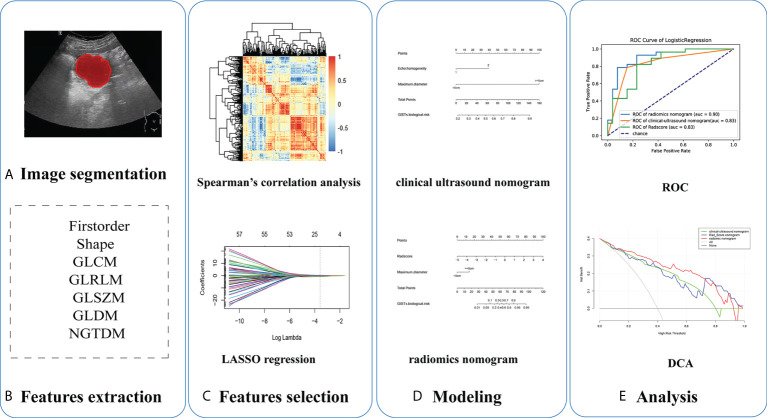
Five steps of radiomics research: **(A)** ultrasound imaging and tumor segmentation, **(B)** image processing and feature extraction, **(C)** feature selection, **(D)** modeling, and **(E)** performance of models.

Research on radiomics mainly includes the following steps: tumor segmentation, image processing and feature extraction, feature selection, modeling, and evaluation (**Figure 2**). In the training cohort, we combined different dimension reduction technologies to establish radiomics models. Finally, the internal validation cohort was used to evaluate the generalization performance of the model.

### Ultrasound image acquisition and tumor image segmentation

Color doppler ultrasonic diagnostic apparatus from Toshiba Aplio 500, Supersonic Aixplorer, and PHILIPS EPIQ5 was used. Transabdominal ultrasound was performed using a convex array probe and a line array probe. We conducted a retrospective review of the image data and selected two-dimensional (2D) ultrasound images in digital imaging and communications in medicine (DICOM) format that scanned by convex array probe, which clearly showed the largest cross-section of each lesion. The tumor location(gastric or extra-gastric),internal echo (hypoecho or isoecho), echo homogeneity (homogeneous or inhomogeneous), boundary (clear or unclear), shape (regular or irregular), blood flow signals of the lesion (according to the Alder blood flow classification, where grades 0 and 1 were merged as low blood supply, and grades 2 and 3 were merged as multiple blood supply), presence of necrotic cystic degeneration (necrotic areas were diagnosed from sharply demarcated anechoic areas), and maximum lesion diameter (≤5.0 cm or >5.0 cm) were recorded in detail.

In this study, the tumor outline at the largest cross-sectional area was manually drawn to indicate the region of interest (ROI) along the tumor margin using a free open-source software package (ITK-SNAP, version 3.8.0, University of Pennsylvania, Philadelphia, USA).

### Evaluation of radiomics feature reproducibility

To assess interobserver reliability and intraobserver reproducibility, 30 cases were chosen randomly from the training cohort for ROI delineation and ROI-based feature extraction by two experienced radiologists (radiologist 1 with an experience of 10 years and radiologist 2 with 5 years’ experience). Interclass and intraclass correlation coefficients (ICCs) were used to evaluate the agreement of feature extraction. The intraobserver ICC was calculated based on two feature extractions by radiologist 1. The interobserver ICC was calculated based on the features extracted first by radiologist 1 and subsequently by radiologist 2. All radiologists were blinded to the pathology results. There was good agreement when the ICC was greater than 0.75, and features were retained with good repeatability (15).

### Radiomics feature extraction and data preprocessing

Because the ultrasound images were collected using three different ultrasound instruments and the feature vectors had a wide range, to eliminate the variance caused by different scanner acquisitions, avoid anisotropic resolution, and improve the reproducibility, we preprocessed the image before feature extraction with the open-source Pyradiomics package (version 2.12; https://pyradiomics.readthedocs.io/en/2.1.2/). Images were normalized by centering to the mean standard deviation,resampled to a voxel size using B-Spline interpolation,and gray-level discretized by a fixed bin width of 5 in the histogram.

The radiomics features of the ROI were extracted using the “PyRadiomics” package in Python. The extracted radiomics features were divided into three categories: 2D shape-based features, first-order statistical features, and structural texture features. The structural texture features include five grayscale matrices: gray-level co-occurrence matrix (GLCM), gray-level run-length matrix (GLRLM), gray-level size-zone matrix (GLSZM), gray-level dependence matrix (GLDM), and features of neighborhood gray-tone difference matrix (NGTDM). Furthermore, to extract high-dimensional features from different frequency scales, eight imaging filters were applied to the raw images: wavelet, square, square root, logarithmic, exponential, gradient, LoG, and local binary pattern (LBP). Finally, 765 quantitative radiomics features were extracted for each patient.

*Z*-score normalization was used to convert different data to the same order of magnitude, and the calculation formula was as follows: *y*=(*x*-*u*)/*σ*, where *µ* is the mean and *σ* is the standard deviation. Following the *Z*-score normalization, the data comparability was improved, demonstrating enhanced robustness for subsequent model building.

### Construction of clinical ultrasound nomogram, calculation of the radiomics score (Rad-Score), and construction of radiomics nomogram

Univariate logistic regression was used to screen statistically significant predictors, and multivariate logistic analysis was subsequently performed for the factors that were determined to be statistically significant. The final model was selected by backward stepwise elimination with Akaike information criteria as the stopping rule and a clinical ultrasound nomogram was constructed (16).

The R software (version 4.0.2) was used for feature dimensionality reduction and construction of the radiomics score (Rad-Score). We followed a three-step procedure to identify robust radiomics features: (1) Feature reproducibility assessment using ICCs was established, and features with high stability (intraclass correlation coefficient >0.75) were retained for further analysis. (2) The high-stability radiomics features were subjected to Spearman’s correlation analysis, with a correlation coefficient threshold of 0.75 (17). (3) We used the least absolute shrinkage and selection operator (LASSO) algorithm, with penalty parameter tuning conducted by 10-fold cross-validation, to select a suitable number of non–zero-weighted features. Rad-Score was calculated for each patient as a linear combination of selected features weighted by the respective nonzero coefficients.

The Rad-Score, combined with clinical and ultrasound factors, was incorporated into multivariate logistic regression to construct a radiomics nomogram.

### Predictive performance and validation of clinical ultrasound nomogram, Rad-Score and radiomics nomogram

All models were validated using an internal validation cohort. The prediction performances of the clinical ultrasound nomogram, the Rad-Score and radiomics nomogram were evaluated by the area under the receiver operating characteristic curve (AUC) for both the training and internal validation cohorts, and accuracy, sensitivity, specificity, negative predictive value (NPV), and positive predictive value (PPV) were calculated. A calibration curve was plotted to assess the calibration of the nomogram using the Hosmer-Lemeshow goodness-of-fit test. P>0.05 indicated insignificant deviation from the theoretical perfect calibration. The clinical application value of the nomogram was determined through decision curve analysis (DCA) by quantifying the net benefit to the patient under different threshold probabilities.

### Statistical analysis

All data were analyzed using the SPSS Statistics software version 23.0 (IBM, Armonk, NY, USA) and R software version 4.0.2 (http://www.Rproject.org).

An independent t-test was applicable to the continuous variables between groups, while a x^2^ test was used for the classification variables between groups. P<0.05 was considered a statistically significant difference.

## Results

### Characteristics of the study cohorts

In this study, 216 GIST patients, comprising 98 with low-malignant potential and 118 with high-malignant potential, were enrolled. There were no significant differences in the distribution of clinical, ultrasound, and radiomics features between the low-malignant and high-malignant potential groups in the training and validation cohorts (P>0.05) (**Table 1**). These results demonstrate the rationality of our training and validation cohort partitions.

**Table 1:** Baseline Patient Characteristics.

Characteristics		Training cohort (n = 163)	Validation cohort (n = 53)	P- value
Patient demographics				
Sex	Male	95 (58.3)	31 (58.5)	0.979
	Female	68 (41.7)	22 (41.5)	
Age(mean ± SD)		60 ± 12	59 ± 11	0.458
BMI(mean ± SD)		23.2 ± 3.1	23.0 ± 3.4	0.787
Ultrasound features				
Tumor location	Gastric	116 (71.2)	35 (66.0)	0.480
	Extra-gastric	47 (28.8)	18 (34.0)	
Maximum diameter	≤5.0cm	86 (52.8)	28 (52.8)	0.993
	>5.0cm	77 (47.2)	25 (47.2)	
The internal echo	Hypoecho	152 (93.3)	51 (96.2)	0.429
	Isoecho	11 (6.7)	2 (3.8)	
Echo homogeneity	Homogenerouse	71 (43.6)	23 (43.4)	0.984
	Inhomogenerouse	92 (56.4)	30 (56.6)	
Boundary	Clear	147 (90.2)	41 (77.4)	0.160
	Unclear	16 (9.8)	12 (22.6)	
Shape	Regular	77 (47.2)	25 (47.2)	1.0
	Irregular	86 (52.8)	28 (52.8)	
Necrotic cystic degeneration	Positive	66 (40.5)	20 (37.7)	0.722
	Negative	97 (59.5)	33 (62.3)	
Blood flow signals	Low blood supply	121 (74.2)	39 (73.6)	0.925
	Multiple blood supply	42 (25.8)	14 (26.4)	
Growth pattern	Endoluminal	82 (50.3)	28 (52.8)	0.750
	Exophytic	81 (49.7)	25 (47.2)	
Radiomics score*		0.51 (-0.77 to 1.12)	0.35 (-0.87 to 1.11)	0.612

Except where indicated, data are numbers of patients, with percentages in parentheses.

SD: standard deviation.

*Data are presented as medians with interquartile ranges in parentheses.

P<0.05 indicates that the difference is statistically significant.

### Establishment and evaluation of clinical ultrasound nomogram

Univariate analysis of clinical and ultrasound parameters showed that there were no statistically significant differences in age, gender, BMI, internal echo, or growth pattern between low-malignant and high-malignant potential groups (P>0.05). However, maximum diameter, tumor location, echo homogeneity, boundary, shape, necrotic cystic degeneration, and blood flow signals were statistically different (P<0.05). These significant clinical and ultrasound features in the univariate analysis were included in the multivariate logistic regression to build a clinical ultrasound nomogram (**Figure 7**). Multivariate logistic regression analysis showed that the maximum tumor diameter (≤5.0 cm, >5 cm) and echo homogeneity (both P< 0.05) were independent predictors of GIST risk, as shown in **Table 2**. The GIST risk prediction results are listed in **Table 5**. The AUC for the clinical ultrasound nomogram was 0.86 in the training cohort and 0.83 in the validation cohort (**Figure 8**).

**Table 2:** Positive clinical and ultrasound factors of multivariate logistic regression analysis for GISTs.

Clinical and ultrasound factors	β	OR (95%CI)	P-value
Intercept	-2.48		
Maximum diameter	2.59	13.34 (6.03-31.59)	0.000
Echo homogeneity	1.01	2.73 (1.23-6.12)	0.000

95%CI, 95% confidence interval; OR, Odds ratio.

### Feature selection and radiomics score building

**Figure 3:**
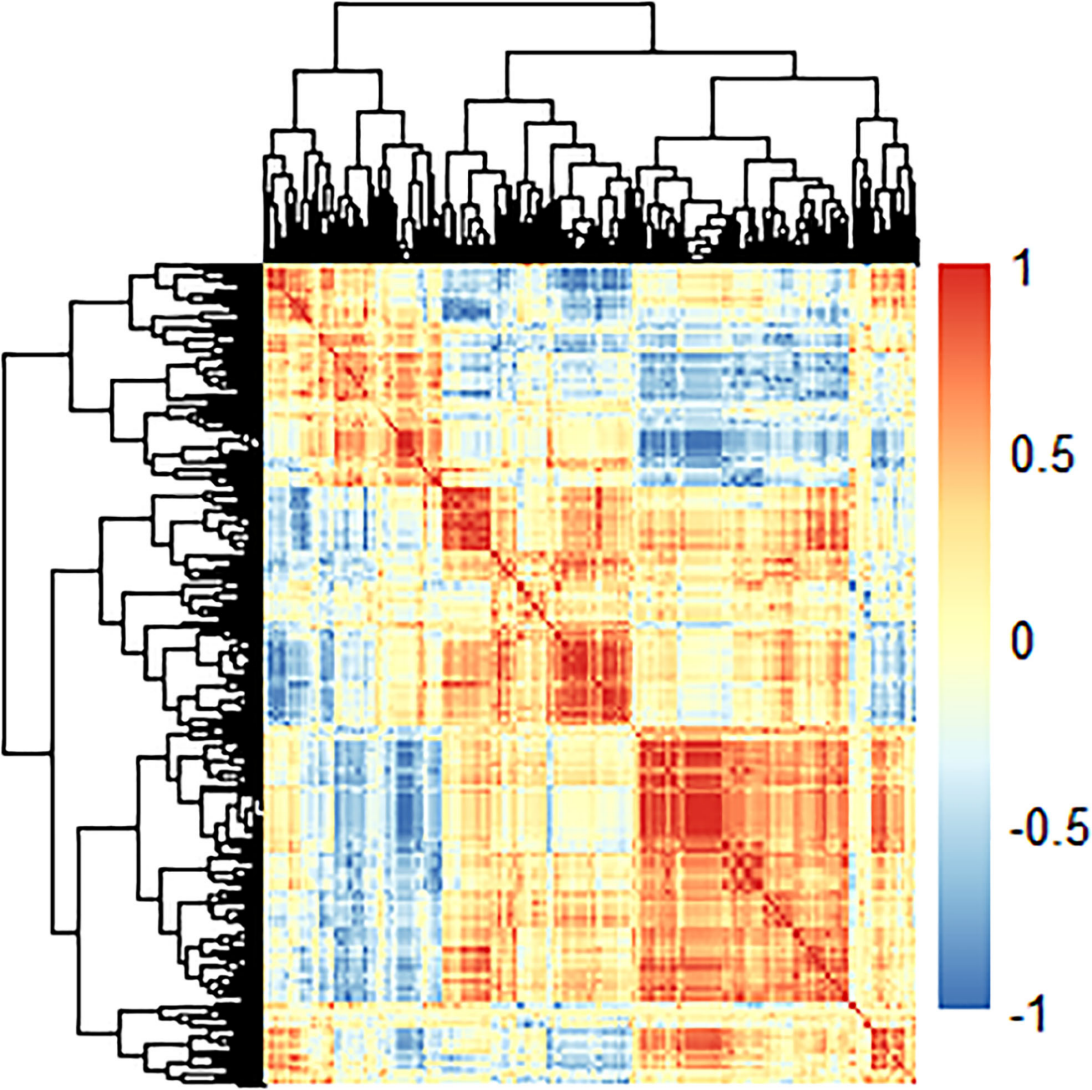
The heat-map displays the correlation between radiomics features in the training cohort.

**Figure 4:**
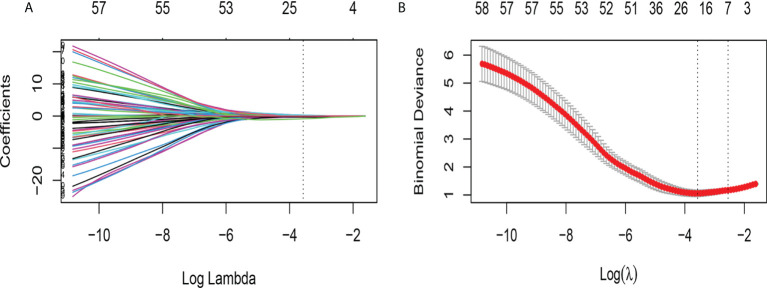
Feature selection using the LASSO binary logistic regression model. **(A)** LASSO coefficient profiles, displaying 68 features. A coefficient profile plot was produced against the log (lambda) sequence. Each colored line represents the coefficient of an individual feature. A vertical line is drawn at the selected λ, where 16 features had nonzero coefficients. **(B)** Tuning parameter (log lambda) selection in the LASSO model used ten-fold cross-validation *via* minimum criteria. Vertical dotted lines are drawn at the selected λ values, corresponding to the chosen 16 variables that better fit the models.

765 radiomics features were extracted. Using an ICC of 0.75 (for both intra and inter) as a cut-off for determining good reproducibility, 612 radiomics features with good reproducibility were selected for the subsequent assessment. After applying Spearman’s correlation analysis (**Figure 3**), 68 features remained. Finally, the LASSO algorithm (**Figure 4**) with cross-validation was performed, and 16 radiomics features were used to develop the Rad-Score. The 16 selected radiomics features and the distribution of the corresponding risk coefficients are presented in **Table 3**. The Rad-Score was calculated using the following formula (18):


$$\text{Radiomics score}=\sum_{i=1}^{n}Coef_{i}\times \text{χi}$$

**Table 3:** Radiomics features and their coefficients that were included in the final assessment of GIST risk.

parameter		Coefficient
Feature1	original_shape2D_Sphericity	-0.37
Feature2	gradient_glcm_Imc1	0.30
Feature3	logarithm_ngtdm_Strength	-0.29
Feature4	square_glcm_Imc1	-0.23
Feature5	square_glrlm_ShortRunLowGrayLevelEmphasis	0.25
Feature6	squareroot_glcm_ClusterShade	-0.25
Feature7	wavelet.LH_glcm_ClusterShade	-0.04
Feature8	wavelet.LH_glcm_Correlation	-0.26
Feature9	wavelet.LH_glcm_MCC	-0.04
Feature10	wavelet.LH_gldm_DependenceVariance	-0.01
Feature11	wavelet.LH_glszm_ZoneEntropy	0.29
Feature12	wavelet.HL_firstorder_Kurtosis	-0.06
Feature13	wavelet.HL_firstorder_Mean	-0.32
Feature14	wavelet.HL_glcm_Imc2	-0.08
Feature15	wavelet.HL_glcm_MCC	-0.10
Feature16	wavelet.HL_gldm_SmallDependenceEmphasis	-0.85

where *Coef_i_* is the risk coefficient of each feature calculated by the LASSO model, and χ_i_ is the expression value of each feature, which refers to the 16 selected radiomics features in the present study. The cross-correlation matrices (**Figure 5**) showed that there were multiple complex cross-correlations among the 16 radiomics features, and Rad-Score indicated favorable prediction for discriminating the risk stratification of GIST (**Figure 6**). There was a significant difference in Rad-Score between the low-malignant and high-malignant potential groups in both the training cohort (−0.696 VS 0.983, P< 0.0001) and the validation cohort (−0.650 vs. 0.952, P< 0.0001).

**Figure 5:**
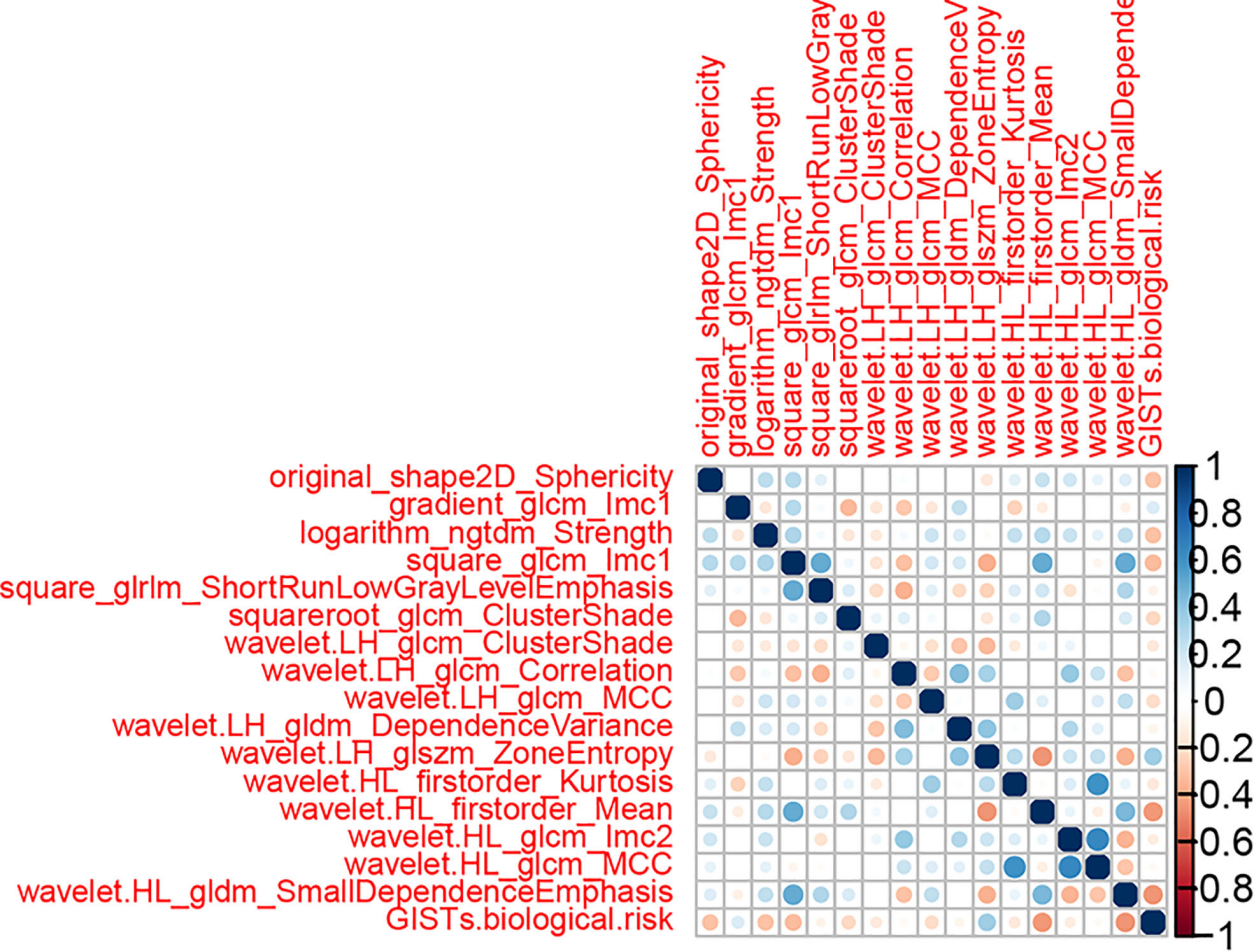
Cross-correlation matrix of features used to establish radiomics signatures. The depth of color indicates the intensity of the correlation between covariates. A cross indicates irrelevant. Blue represents positive correlations, and red represents negative correlations.

**Figure 6:**
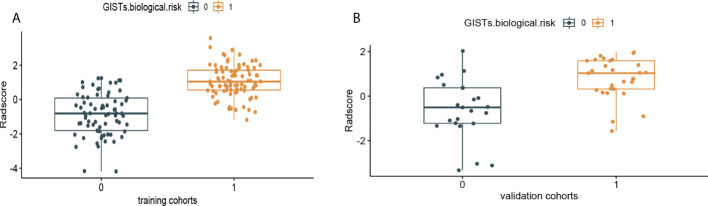
Rad-Score distribution of the 216 patients for **(A)** the training cohort and **(B)** validation cohort. The low-malignant potential group is colored blue (0), and the high-risk group is colored yellow (1).

The prediction performance for discriminating the risk stratification of GIST of the Rad-score (AUC, 0.90 in the training cohort and 0.83 in the validation cohort) was not remarkable difference that of clinical ultrasound nomogram in the training cohort (p=0.193) and validation cohort (p = 0.947) (**Table 5**).

### Development and validation of the radiomics nomogram

**Figure 7:**
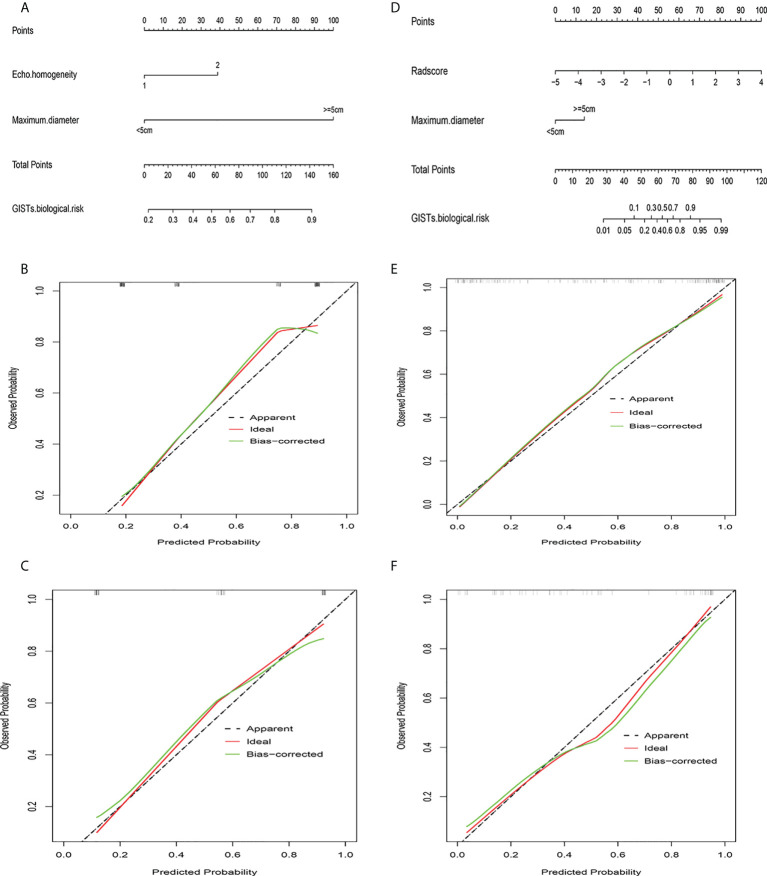
Clinical ultrasound nomogram and radiomics nomogram. **(A)** Nomogram of clinical ultrasound model. **(B)** Calibration curve of clinical ultrasound nomogram in training cohort. **(C)** Calibration curve of clinical ultrasound nomogram in validation cohort. **(D)** Nomogram of radiomics model. **(E)** Calibration curve of radiomics nomogram in training cohort. **(F)** Calibration curve of radiomics nomogram in validation cohort.

**Figure 8:**
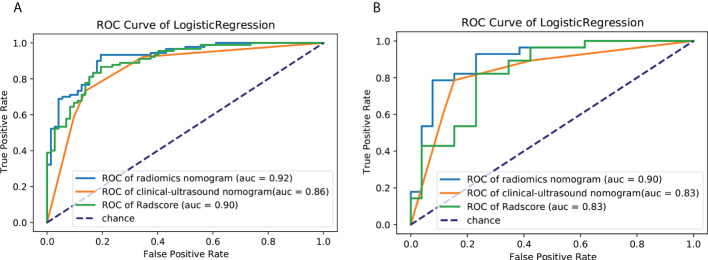
ROC curves of the clinical ultrasound nomogram,the Rad-Score and radiomics nomogram for predicting malignancy potential of GISTs in the **(A)** training cohort and **(B)** validation cohort. AUC, area under the receiver operating characteristic curve; GIST, gastrointestinal stromal tumor.

**Figure 9:**
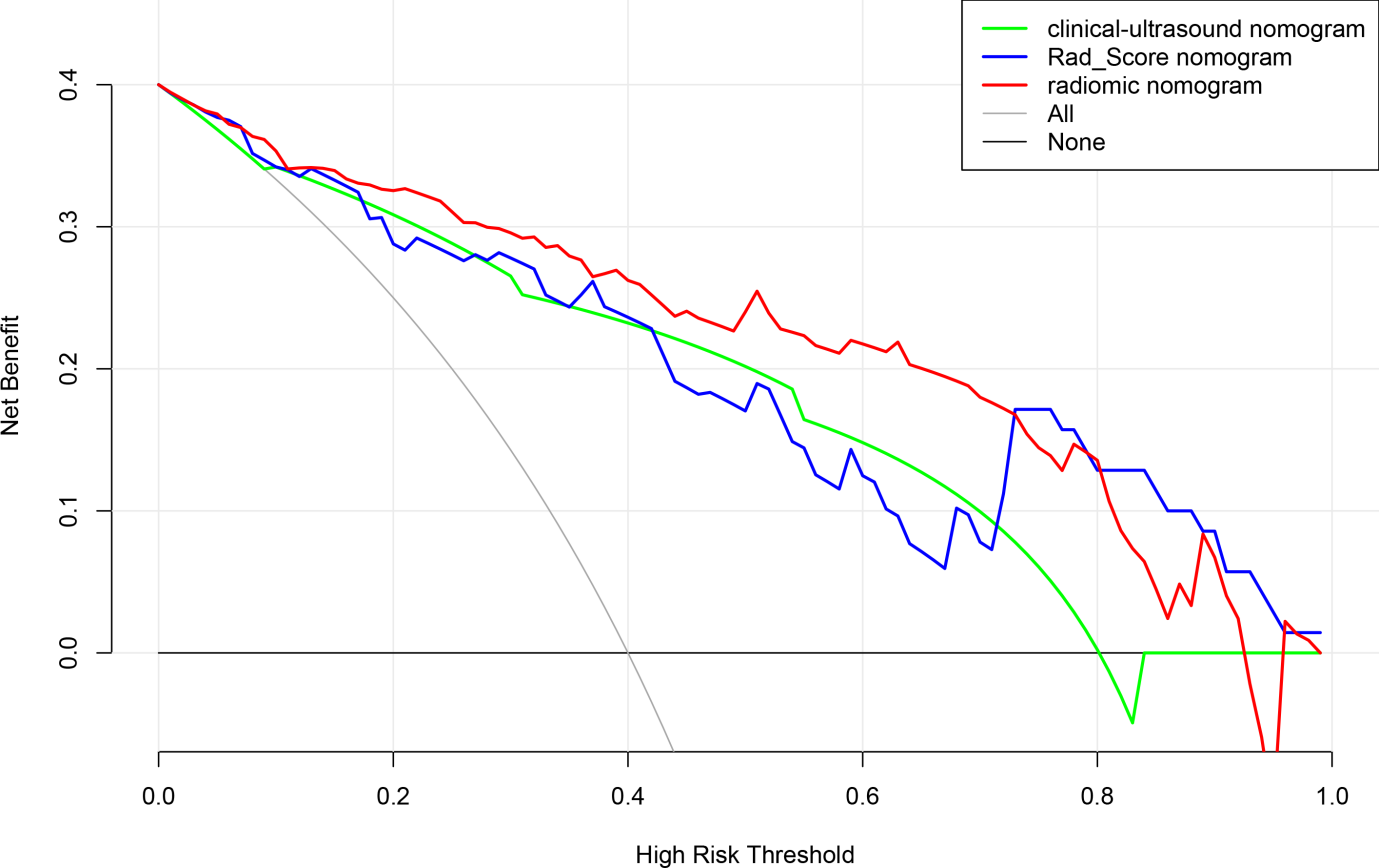
Decision curve analysis for clinical ultrasound nomogram,the Rad-Score and radiomics model nomogram.

According to the results of the multivariate logistic regression analysis (**Table 4**), a radiomics nomogram was generated by combining clinical ultrasound features and Rad-Score (**Figure 7**). The AUCs of the radiomics nomogram for predicting the GIST risk were 0.92 and 0.90 (training and validation cohorts, respectively), and its predictive accuracy was higher than that of the clinical ultrasound nomogram (P = 0.011)or the Rad-score (P = 0.018)in the validation cohort(**Table 5**, **Figure 8**). Of the 114 tumors smaller than 5 cm, six were underestimated by the clinical ultrasound nomogram, and 3 of the 102 tumors larger than 5 cm were overestimated by the clinical ultrasound nomogram, but these were correctly assessed by the radiomics nomogram. The radiomics nomogram calibration curves showed good calibration (**Figure 7**), and the Hosmer-Lemeshow test statistics were not significant in either the training or internal validation cohorts, indicating that the radiomics nomogram provided a higher net benefit than the clinical ultrasound nomogram or the Rad-score within a reasonable threshold probability range for predicting the malignant potential of GISTs (**Figure 9**).

**Table 4:** Variables and coefficients of the radiomics nomogram by multivariate logistic regression analysis.

Variable	β	OR (95%CI)	P-value
Intercept	-0.90		
Maximum diameter	1.88	6.57(2.54-18.0)	0.000
The Rad-Score	1.49	4.43(2.68-8.21)	0.000

95% CI, 95% confidence interval; OR, Odds ratio.

**Table 5:** Predictive performance of the radiomics nomogram compared with clinical ultrasound nomogram and the rad-score.

Model		AUC	Accuracy	Sensitivity	Specificity	PPV	NPV
Clinical ultrasound nomogram	training cohort	0.86	0.80	0.75	0.86	0.86	0.74
	validation cohort	0.83	0.83	0.88	0.79	0.84	0.82
The Rad-Score	training cohort	0.90	0.83	0.87	0.79	0.83	0.86
	validation cohort	0.83	0.79	0.90	0.57	0.74	0.81
radiomic nomogram	training cohort	0.92	0.84	0.84	0.85	0.87	0.81
	validation cohort	0.90	0.88	0.89	0.87	0.90	0.88

AUC, area under the receiver operating characteristic curve; PPV, positive predictive value; NPV, negative predictive value.

## Discussion

As a rapidly developing discipline, radiomics is being increasingly used for clinical applications. Radiomics can extract feature data from digital medical images in a high-throughput manner, and this data can be quantitatively analyzed for the transformation of conventional clinical data into mineable high-dimensional quantitative feature data. In this study, radiomics features were combined with clinical and ultrasound image features to establish a radiomics-based nomogram for predicting the malignant potential of GISTs. Our results confirm that the radiomics nomogram has greater predictive power than the clinical ultrasound nomogram or the Rad-score for the malignant potential of GISTs and can guide clinical decision-making before surgery noninvasively and effectively.

According to previous studies, clinical and ultrasound features can help sonographers intuitively diagnose and predict the risk of GISTs (19, 20). Ultrasound imaging features mainly include the location, size, shape, growth pattern, internal echo, and cystic necrosis of the lesion (21). In this study, 12 variables of clinical and ultrasound signs were collected, and a clinical ultrasound model was established through univariate and multivariate logistic regression. Finally, two variables— tumor size and tumor echo homogeneity— were incorporated into the model, and a nomogram of the clinical ultrasound model was drawn. Tumor size is an important factor in evaluating the malignant potential and prognosis of GISTs. It is listed as a risk stratification index by the NIH grading system. The larger the tumor size, the higher the risk of infiltration (22–24). Nevertheless, evaluating the malignancy of GISTs based on tumor size alone is insufficient. According to the NIH standards, the risk grade of GISTs is not only related to tumor size, but also to the mitotic index, tumor location, and whether or not the tumor is ruptured. Some small GISTs that are located in the intestine and possess high mitotic counts may be aggressive and have poor prognosis.

Echo homogeneity of GISTs is also an independent predictor of clinical ultrasound nomograms for evaluating the biological behavior of GISTs, which may be related to the fact that with increasing tumor malignancy, cystic degeneration and necrosis are more likely to occur inside the mass when the rate of differentiation and proliferation of tumor cells far exceeds the rate of proliferation of blood vessels (25, 26).

In ultrasonography, cystic degeneration and necrosis appear as an intratumoral inhomogeneous echogenicity. Previous studies (27–30) have shown that the presence of cystic degeneration and necrosis in the mass can be used as reliable indicators for GISTs malignancy evaluation, which is consistent with the results of our study. In this study, the AUC of the clinical ultrasound nomogram for predicting the malignant potential of GISTs was 0.86 in the training cohort and only 0.83 in the validation cohort. Although tumor size and echo homogeneity are important variables for predicting the risk stratification of GISTs, they cannot fully reflect the inherent heterogeneity of GISTs and are easily affected by subjective factors and the professional level of sonographers.

Radiomics features extracted from ultrasound images can assess intratumor heterogeneity by quantifying tumor morphology, intensity, and texture features (31, 32), which could more effectively and objectively predict the malignant potential of GIST preoperatively. In this study, 765 radiomics features were extracted from ultrasound images using pyradiomics software. There was serious multicollinearity among the feature parameters. Features were extracted by the Spearman and LASSO algorithm methods to reduce overfitting. As a new feature selection method, Spearman and Lasso can achieve less redundancy and obtain more reliable radiomics features. The selected 16 radiomics features included one 2D shape-based feature, two first-order statistical features, and 13 texture features. These radiomics features represent an assessment of tumor shape and tumor heterogeneity (33).

Sphericity (measured as the shape of the ROI) is the ratio of the perimeter of the tumor region to the perimeter of a circle with the same surface area as the tumor region, which is correlated with the biological risk of GISTs. The first-order statistical features indicate that the image texture is disordered and complex, containing more information, and the pixel distribution is more random. High-order texture features reflect comprehensive information, such as space and distance, which can supplement the deficiencies of first-order statistical features (34). The radiomics features screened from GIST lesions in this study were significantly positively correlated with risk stratification (**Figure 7**), which indicates that high-malignant potential tumors have complex internal textures and random distributions. This hypothesis may be because of the fact that high-malignant potential tumors are usually large, fast-growing, have insufficient blood supply to the central tissue, and are more prone to necrosis, rupture, and hemorrhage. Therefore, in our study, the Rad-Score based on multiple features was an important factor for assessing information regarding tumor heterogeneity.

When the Rad-Score was combined with clinical and ultrasound factors to incorporate multiple logistic regression, the results demonstrated that the tumor size and Rad-Score are independent predictors of the malignant potential of GISTs, thereby constructing a radiomics nomogram. Radiomics can quantitatively analyze the image characteristics and more accurately reflect tissue heterogeneity. The radiomics nomogram showed higher predictive performance (training cohort AUC: 0.92; validation cohort: 0.90) than the clinical ultrasound nomogram or the Rad-Score. In our retrospective data, 6 cases of high-malignant potential with tumor size less than 5 cm were accurately diagnosed using the radiomics nomogram, but they were underestimated by the clinical ultrasound nomogram. This suggests that our radiomics nomogram has better predictive performance in some cases with small tumor size. When the tumor size was larger than 5 cm, the total number of misclassifications in the clinical ultrasound nomogram remained higher than that in the radiomics nomogram. Therefore, the use of nomograms to further integrate radiomics features with these subjective clinical and ultrasound image features can lead to better diagnostic performance with good calibration. In this study, a radiomics-based nomogram provided a more accurate and personalized prediction for preoperative risk assessment of patients with GISTs by combining clinical and ultrasound risk factors. Additionally, the decision curves showed that the radiomics nomogram was superior to the clinical ultrasound nomogram or the Rad-Score in terms of clinical validity, which confirmed the potential of the radiomics nomogram for broader clinical applications.

This study developed the first radiomics model based on 2D transabdominal ultrasound images to predict the risk stratification of GISTs. However, this study has some limitations. First, all data were obtained from a single center, and a multicenter study must be designed for further evaluation and validation. Second, this was a retrospective study, and selective bias could not be completely avoided. Third, we did not compare the feature extraction and dimensionality reduction algorithms, and the final feature selection may not be optimal, which may affect the predictive performance of the model.

## Conclusion

As a noninvasive, non-radiation, and objective method, a radiomics nomogram based on radiomics features from 2D ultrasound and maximum tumor diameter was constructed for predicting malignant potential in GISTs, which could provide supplementary information for the prognostic evaluation of GISTs and guide patients to select the best treatment method minimizing their medical burden.

## Data availability statement

The original contributions presented in the study are included in the article/supplementary material. Further inquiries can be directed to the corresponding author.

## Ethics statement

The studies involving human participants were reviewed and approved by the ethics committee of Fujian Medical University Affiliated Union Hospital. Written informed consent for participation was not required for this study in accordance with the national legislation and the institutional requirements.

## Author contributions

ZC proposed the conception and design of this research and analyzed and interpreted the data. MZ and JG developed methodology. YT, XT, and QQ collected data and performed preprocessing. MZ, JG, and ZC were major contributors in writing the manuscript. All authors contributed to the article and approved the submitted version.

## Funding

This work was supported by the 5th Round Joint Research Project from the Health and Family Planning commission and Education Department of Fujian Province of China under Grant (2019-WJ-06).

## Conflict of interest

The authors declare that the research was conducted in the absence of any commercial or financial relationships that could be construed as a potential conflict of interest.

## Publisher’s note

All claims expressed in this article are solely those of the authors and do not necessarily represent those of their affiliated organizations, or those of the publisher, the editors and the reviewers. Any product that may be evaluated in this article, or claim that may be made by its manufacturer, is not guaranteed or endorsed by the publisher.
